# Japanese beetles’ feeding on milkweed flowers may compromise efforts to restore monarch butterfly habitat

**DOI:** 10.1038/s41598-018-30731-z

**Published:** 2018-08-14

**Authors:** Adam M. Baker, Daniel A. Potter

**Affiliations:** Department of Entomology, University of Kentucky, Lexington, KY USA

## Abstract

The eastern North American migratory population of monarch butterflies (*Danaus plexippus*) is in serious decline. Habitat restoration, including adding millions of host plants to compensate for loss of milkweed in US cropland, is a key part of the international conservation strategy to return this iconic butterfly to sustainable status. We report here that *Popillia japonica*, a polyphagous, invasive beetle, aggregates and feeds on flowers of *Asclepias syriaca*, the monarch’s most important larval food plant, reducing fruiting and seed set by >90% and extensively damaging milkweed umbels in the field. The beetle’s ongoing incursion into the monarch’s key breeding grounds in the US Midwest is likely to limit pollination and outcrossing of wild and planted milkweeds, reducing their capacity to colonize new areas via seeds. *Popillia japonica* represents a previously undocumented threat to milkweeds that should be considered in models for monarch habitat restoration.

## Introduction

The eastern migratory population of the monarch, *Danaus plexippus* L., probably the best known butterfly in the world, has declined in abundance by > 90% in the last two decades^1^ and is considered at risk of extirpation^2,3^. The monarch has become an international conservation icon with power to mobilize scientists, organizations, and the public into actions to help restore its populations, and shape environmental policy^4–7^. Conservation of this specialist herbivore requires understanding the threats affecting its annual abundance, one of which is loss of milkweed (*Asclepias* species), the essential larval host plants, in the monarch's summer breeding grounds in the Midwestern United States^8–12^. We report here a previously undocumented biotic threat to sexual reproduction of common milkweed, *Asclepias syriaca*, which is used by >90% of monarchs in their summer breeding range within eastern North America^13–17^.

**Figure 1:**
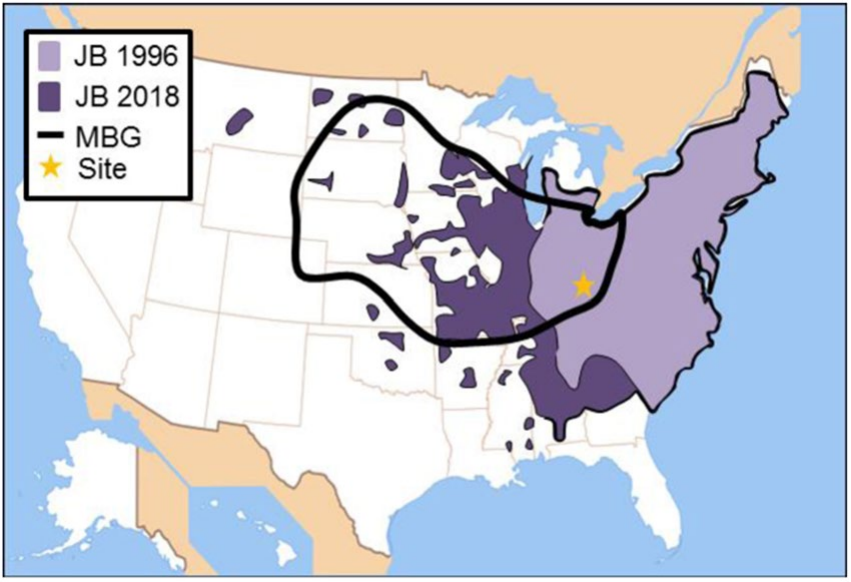
Japanese beetle [JB] incursion into the monarch butterfly breeding grounds [MBG] of the US Midwest. JB distributions are based on USDA APHIS Cooperative Agricultural Pest Survey maps *(19)*. Light purple denotes areas occupied by JB in 1996; dark purple denotes additional areas where JB had become established by 2018. Black line encloses the geographic region of the United States that is estimated, based on stable isotope analysis and geospatial modeling, to have produced the highest proportion of monarchs overwintering in Mexico over a 38-year period from 1976–2014^20^. Star represents the location where the research described herein was conducted.

*Popillia japonica* Newman, commonly known as the Japanese beetle [JB], is an invasive, polyphagous scarab that was first discovered in Riverton, New Jersey, USA, near Philadelphia, in 1916^18^. Until then the species had not been known to inhabit North America. It is now widely established in the eastern United States and SE Canada, but is still expanding in abundance and range in the US Midwest^18,19^. The JB’s distribution now overlaps much of geographic region that, relative to other regions, has produced the highest proportion of monarch butterflies overwintering in Mexico over the past four decades^20^ (Fig. 1).

**Figure 2:**
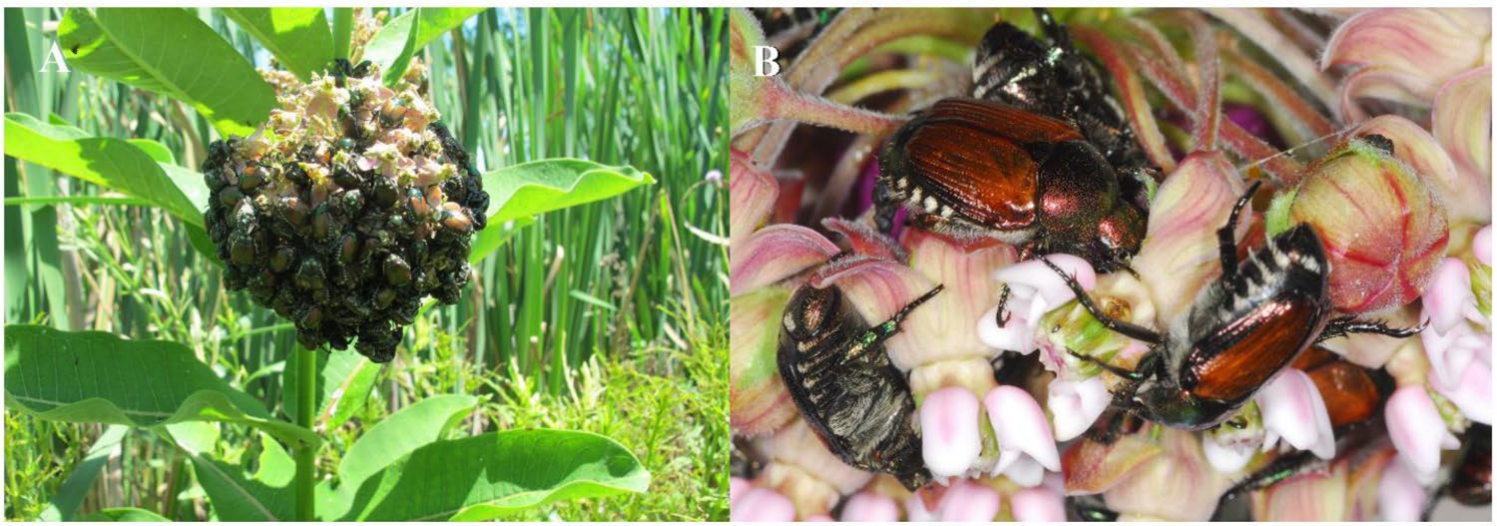
Japanese beetle [JB] feeding on common milkweed, *Asclepias syriaca*. (**A**) Aggregation of 288 JB on milkweed umbel (inflorescence). Infestations and florivory were widely observed in 2016–2018 and occurred on >90% of surveyed plants. **(B)** JB biting into coronal hoods of individual flowers.

During routine surveys for monarch butterfly larvae, we observed large feeding aggregations of JB on umbels (large round inflorescences of 30–75 or more flowers) of *A*. *syriaca* growing wild in pasture land, naturalized areas of parks, and other settings in central Kentucky (Fig. 2A). The beetles were observed using their mandibles to remove the coronal hoods (saccate extensions of staminal tissue in which nectar is stored) from individual flowers to expose the nectaries and other floral structures (Fig. 2B). Here we verify the extent of JB aggregation on milkweed and damage to umbels in wild stands of milkweed, clarify which stage of bloom and floral parts the JB prefers to feed upon, and assess the impact of JB florivory on fruit and seed set of *A*. *syriaca* umbels in the field.

## Results

### Extent of infestation

**Figure 3:**
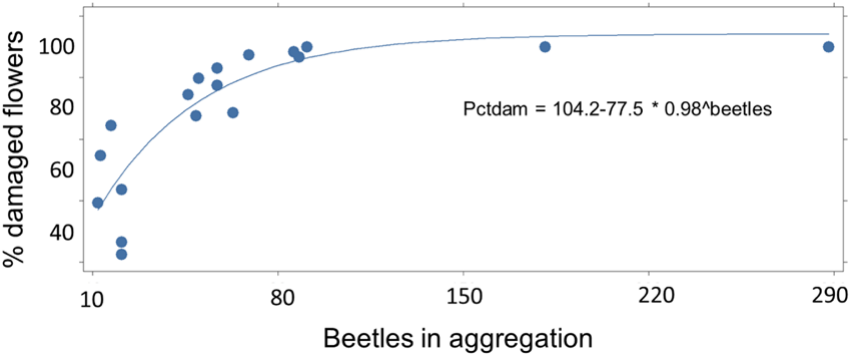
Non-linear regression fitted curve showing asymptotic relationship between number of Japanese beetles in natural aggregations on *A*. *syriaca* umbels and percentage of flowers already damaged. At the time of collection, aggregations of 40 or more JB had destroyed 75–100% of the individual flowers.

A census to gauge extent of JB florivory on *A*. *syriaca* at three periurban field sites in central Kentucky revealed beetle aggregations and feeding damage to umbels on 98% (98/100), 90% (180/200), and 93% (185/200) of 500 total plants. Extent of floral damage was assessed by bagging 18 umbels with naturally-occurring aggregations in the field, removing and counting the beetles, and then dissecting the umbels and examining individual flowers under a binocular microscope. Aggregation size ranged from 12 to 288 JB per umbel (mean ± SE: 68 ± 16), with an asymptotic relationship between aggregation size and percentage of damaged flowers (Fig. 3). Sex ratio within aggregations was male-biased (mean ± SE: 57.1 ± 4.5% males; range: 41.2–77.8%, n = 8). Females were mostly feeding, whereas males often were mounted on females or other males and not feeding.

### Stage of bloom and floral parts preferred

To clarify how flower bud development affects susceptibility to JB feeding, we collected similar-sized umbels in different stages of bloom (closed green bud, pink bud, or with open flowers; see Supplementary Fig. S1), confined them individually with five female JB per umbel, and evaluated numbers of buds or flowers that were damaged. After 24 h, the JB had damaged 1.7 ± 1.1, 11.6 ± 9.3, and 45.1 ± 8.0% of the individual buds or flowers on umbels of those developmental stages, respectively (*F*_2,9_ = 11.7; *P* < 0.005).

Milkweeds are remarkable in their floral complexity and means by which pollination is accomplished^21^. Nectar is secreted within the five stigmatic chambers formed by stiffened, wing-like elaborations of the adjacent anthers, and stored within saccate extensions of staminal tissue, the hoods, which together form the corona. Each pair of adjacent anther wings forms a slit that allows access to the stigmatic chamber. Two sac-like pollinia (masses of pollen) are located on either side of the stigmatic chamber and joined together at the top of the stigmatic slit. When a nectar-seeking insect visits a donor flower, a leg may become caught in a stigmatic slit, dislodging the paired pollinia that become stuck to the pollinator’s appendage or body hairs. When the insect visits another plant of the same milkweed species, a pollinium may be inadvertently inserted into the stigmatic chamber of a recipient flower. Successful pollination results in enlargement of one of the carpels, producing a fruit (pod) containing numerous seeds.

**Figure 4:**
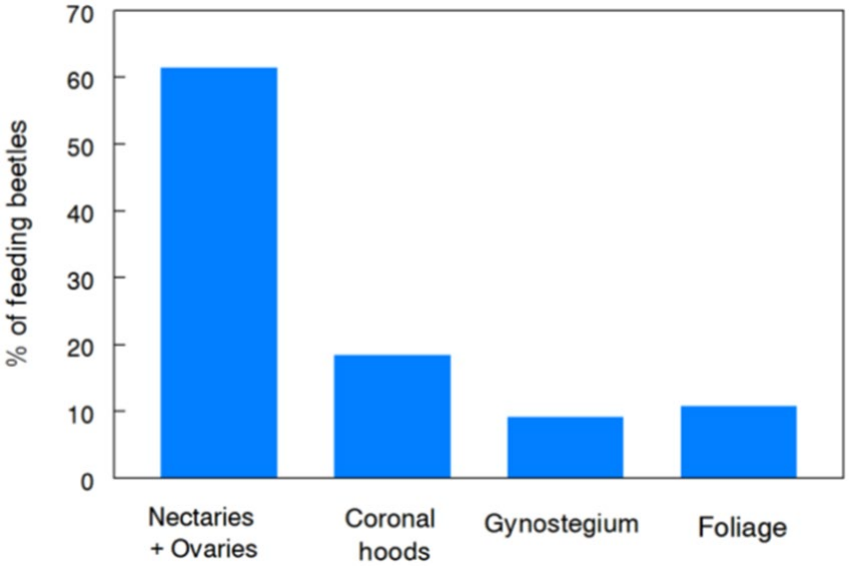
Frequency distribution of Japanese beetles [JB] feeding on floral organs or foliage of *A*. *syriaca* in choice tests. Flowers were dissected into component parts: nectaries + ovaries (on pedicel), coronal hoods, gynostegium (stigmatic chambers + pollinaria) and offered to individual females (n = 80) in four-way choice tests that included a 1 cm^2^ piece of leaf tissue. Food choice of JB that fed (n = 65) differed significantly from the null hypothesis of no preference (χ^2^ = 47.6, df = 3, *P* < 0.001).

To clarify which part(s) of the flower that JB prefers feeding on, we separated 80 individual flowers into their component parts^21^: coronal hoods, nectaries + ovaries (on pedicel), or gynostegium (stigmatic chambers + pollinaria) which were offered to individual females in four-way choice tests that also included a 1-cm^2^ piece of leaf tissue. The nectaries + ovaries were strongly preferred (Fig. 4).

### Effects of JB florivory on fruit and seed set

**Figure 5:**
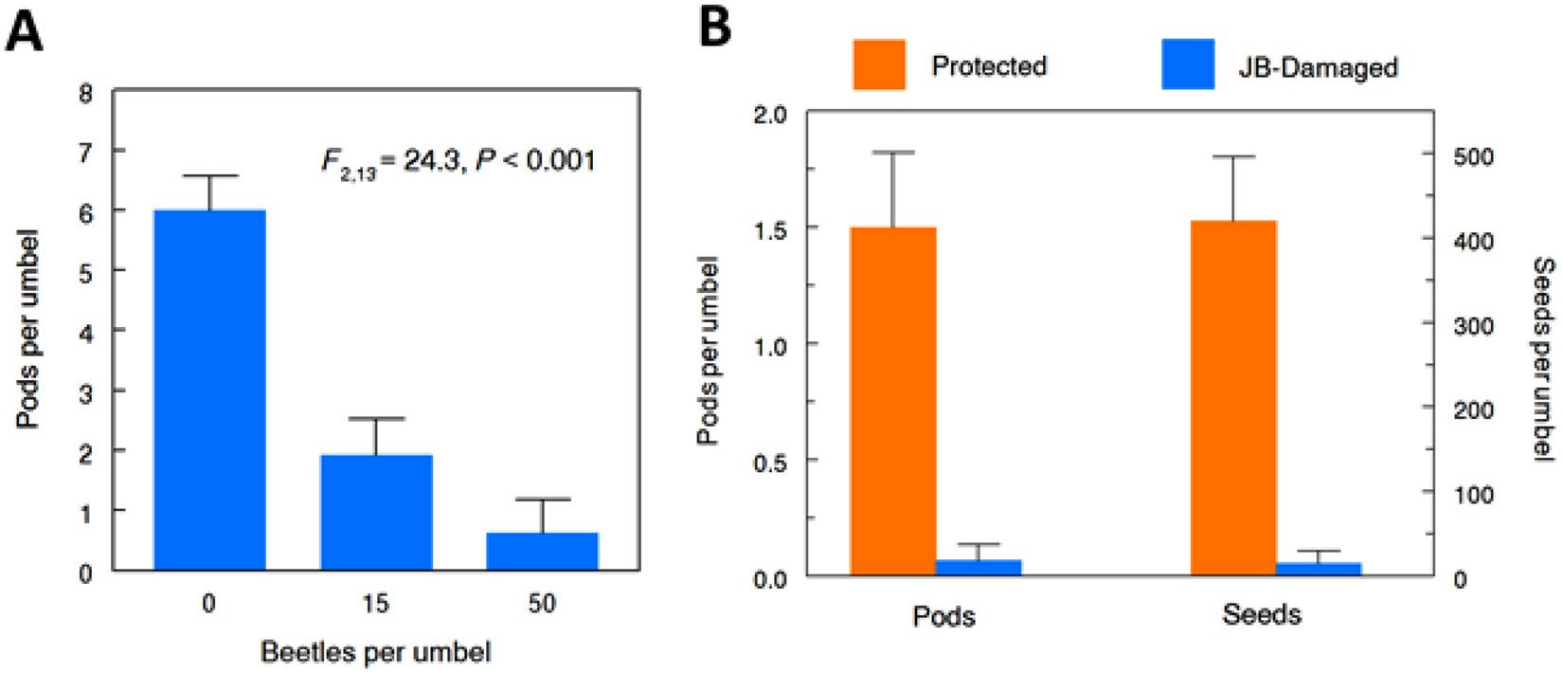
Japanese beetle [JB] feeding on umbels reduces milkweed fruiting and seed set. (**A**) Field-realistic densities of JB caged on intact umbels for 24 h reduced early fruit set. (**B**) Damage from natural JB aggregations greatly reduced numbers of mature pods and seeds (Wilcoxon rank sum test, *P* < 0.001). Bars represent means + standard error.

Field-realistic densities of JB (0, 15, or 50 per umbel) were caged in mesh bags (Supplementary Fig. S2) on undamaged umbels of common milkweed in natural stands (eight replicates per density on separate plants) and allowed to feed for 24 h, after which the JBs were removed and the bags were replaced to prevent further florivory and left until formation of pods (fruits). Compared to the controls, just one days’ feeding by 15 or 50 JB reduced initial pod set by 67 and 90%, respectively (Fig. 5A).

The trial was repeated, except this time we bagged umbels with or without natural JB aggregations (mean: 66.7 ± 9.9 per aggregation; range: 13–147) on separate plants (n = 15 per treatment) in the field, left the bags in place for 24 h, removed the JB, and replaced the bags to shield them from further damage as before. Ten of the 14 surviving shoots upon which the umbels were protected from JB produced mature pods that collectively yielded 5658 total seeds (means: 2.3 ± 0.26 pods per umbel, 246 ± 14 seeds per pod). The fifteen umbels that had been fed upon collectively produced only a single fruit that yielded 223 seeds, representing 96.5% reduction in seed set following JB florivory (Fig. 5B).

## Discussion

Why do JB aggregate and feed on *A*. *syriaca* umbels? The polyphagous, day-flying beetles have high energetic requirements^22^ and they will exploit sugar-rich foods including nectar and floral tissues^18,23,24^. They are attracted to floral odors and aggregate in response to feeding-induced volatiles from damaged plant tissues^25^. Individual milkweed flowers are long-lived (about 5 d for *A*. *syriaca*) and produce copious amounts of high-sucrose nectar^21,26^. Milkweed pollen germinates in nectar secreted within the stigmatic chamber^26^. *Popillia japonica* chew into the stigmatic hoods of individual flowers to rob the nectar and feed on the ovaries, destroying the flowers before or after pollination and preventing formation of fruit and seeds. The beetles sometimes also feed secondarily on milkweed leaves distal to vein cuts made by specialist milkweed herbivores^27^, but the extent of that injury is unlikely to affect plant fitness.

JB florivory on *A*. *syriaca* is not restricted to central Kentucky where the beetle has been abundant for at least 40 years. Similar damage is occurring in other long-infested eastern states, in the US Midwest where the beetle is more recently established, and in the Great Plains at the invasion front (Supplementary Fig. S3). JB populations fluctuate from year to year but because of their affinity for nectar-feeding on *A*. *syriaca*, they are likely to aggregate on milkweed umbels even in “down” years. Endemic generalist predators, introduced parasitoids, and endemic and introduced pathogens collectively help to suppress JB populations but historically have not been effective enough to prevent the beetle’s range expansion, establishment, and damage to favored host plants in North America^18^.

The eastern monarch population faces threats at different locations and times during its multi-generational migration between overwintering sites in the forests of central Mexico and summer breeding grounds in the US and Canada^10,16,17,28^. The recent population decline has been predominantly attributed to loss of overwintering habitat^1^ and shortage of larval host plants and nectar resources in the key breeding grounds of the US Midwest where increased use of herbicides to kill weeds in genetically-altered, glyphosate-tolerant crops has coincided with a dramatic reduction in milkweed abundance^8–10,29,30^. Demographic analyses suggest that conserving and planting milkweed to restore the carrying capacity of the breeding grounds is important for stabilizing the monarch population^9,30,31^.

In 2015, The White House announced a National Strategy to promote the health of pollinators that included restoring by 2020 sufficient habitat in the United States to support an eastern migratory monarch population of 225 million butterflies occupying 6 ha of overwintering habitat in Mexico^5^. Mexico and Canada subsequently adopted that goal as part of a long-term cooperative agenda to conserve the monarch and its unique migratory phenomenon^32^. Planting of milkweed on public and private lands has emerged as a central conservation strategy^15,33,34^.

*Asclepias syriaca*, which is the main larval host plant for monarchs in their summer breeding range in North America accounting for 92% of the butterflies that overwinter in Mexico^13–15^, has been the focus of nearly all initiatives for restoring and enhancing monarch breeding habitat^15,30,35^. The major vectors of *A*. *syriaca* pollinia are Hymenoptera and Lepidoptera, particularly large bees and moths^21,26,36^, and those floral “generalist” pollinators effect extensive gene flow within and between populations, boosted by wind dispersal of comose seeds^21^. Adult JB activity extends from early June to late August^18,37^ coinciding with the entire reproductive window of *A*. *syriaca*. Although the JB is unlikely to reduce survival of individual plants, which can clonally reproduce via rhizomes^21^, its florivory will limit pollination and outcrossing, and decrease milkweed’s capacity to colonize new areas via seeds.

### Conclusion and Implications

The effects of JB florivory on fruit and seed set of milkweed have not been considered in existing estimates^15,30,35^ for how much milkweed must be restored to support the aforementioned conservation goals. Given the JB’s outbreak status in the US Midwest and its continuing expansion in the main monarch flyways^19^, this invasive pest is likely to limit outcrossing and reproduction of wild milkweeds, as well as those planted for monarch habitat restoration. The beetle may also impact the milkweed seed industry that is concentrated in the central Midwest and currently provides most of the seed used for monarch habitat restoration, as well as reproduction of other milkweed species, including a number that are formally designated as threatened or endangered^38^ at state or federal levels.

## Methods

### Extent of JB infestation of *A. syriaca* in the field

Japanese beetle [JB] florivory on wild *A*. *syriaca* was surveyed at two periurban field sites in central Kentucky, a natural-area park consisting of 133 ha of rolling pasture land (Hisle Farm Park; 38°04′27.4″N 84°23′32.7″W), and naturalized areas of a golf course, (University Club of Kentucky; 38°06′49.5″N 84°36′28.7″W), in mid-July 2017. An additional site in naturalized areas of a different golf course (Kearney Hill Golf Links, 38°07′33.2″N 84°32′26.9″W) was sampled in early July 2018. At each site, we walked transects in four locations and scored the incidence of plants with JB aggregations or obvious severe feeding damage on their umbels. The stands of milkweed are naturally occurring at all three sites, and managed by mowing once or twice per year. In addition to milkweed, all sites contained a mix of spontaneous herbaceous plants including tall fescue (*Festuca arundinacea*), knapweed (*Centaurea* sp.), common yarrow (*Achillea millefolium*), clover (*Trifolium* spp.), poison hemlock (*Conium maculatum*), and other species resulting from natural succession into fallow areas. The sites were surrounded by areas of high-mowed (≥9 cm) mixed tall fescue and Kentucky bluegrass (*Poa pratensis*) and bordered by hedgerows with woody plants including black locust (*Robinia pseudoacacia*), black cherry (*Prunus serotina*), hackberry (*Celtis occidentalis*), river birch (*Betula nigra*), and sugar maple (*Acer saccharum*).

### Stage of bloom and particular floral parts preferred

Beetles were field-collected with standard JB traps (Trécé, Adair, OK, USA) baited with food-type lures (2-phenyl-ethyl-propionate, eugenol, and geraniol, 3:7:3 ratio) and brought to the lab within 4 h. Sexes were separated by foretibial characters^37^ and males were discarded. Females were held overnight without food before each assay. Freshly caught beetles were used for each trial.

For the trial clarifying how milkweed bud development affects susceptibility to JB feeding, stems with umbels of three phenological stages (closed green bud, pink bud, or open flowers) were harvested from wild plants, placed in vases with water, and brought to the lab. Umbels were placed in 0.5 liter clear plastic containers with five females and held at 27 °C and 16:8 h (L:D) in a growth chamber for 24 h after which all flowers were excised and examined for feeding damage.

To clarify which floral organs are preferred, we harvested umbels with fully-opened flowers, separated 80 individual flowers into their component parts: coronal hoods, nectaries + ovaries (on pedicel), or gynostegium (stigmatic chambers + pollinaria) *(21)* and offered to individual JB females in four-way choice tests that also included a 1-cm^2^ piece of freshly-cut leaf tissue. Test arenas were translucent plastic containers (11 cm diameter, 4 cm high) with a screened lid. Feeding preference was scored after 20 min.

### Impact of JB on *A. syriaca* fruit and seed set

For trials in which JB were caged on wild plants in the field, mature umbels with beetles were enclosed in light-weight fine mesh secured around the stem using a wire twist tie (Fig. S2). Each umbel was on a different plant. The trials were done at Hisle Farm Park (see above). The trial with manipulated JB densities used females collected with traps and starved overnight as described earlier. The JB were caged on the umbels on 26 June 2017 and removed after 24 h; the umbels were re-bagged and initial pod set was evaluated 30 d later.

For the trial with natural JB aggregations, we located non-infested umbels and ones with a range of JB densities and enclosed them in mesh as above. The plants were spaced at least 3–5 m apart to avoid disturbing the JB before they were bagged. Umbels were caged on 7 July 2017, JB were removed and counted after 24 h, and then umbels were re-bagged to prevent further florivory. Mature pods and seeds were counted on 20 September 2017.

### Statistical analyses

Data were tested for assumptions of normality and homogeneity of variance implicit in parametric tests. Arsine transformation was used on percentage data. The asymptotic regression curve shown in Fig. 3 was fitted using an iterative function minimization algorithm (Levenberg-Marquardt-Nash algorithm) to obtain the least square estimates of the parameters. Analysis of variance was used to compare JB feeding damage between buds and flowers of different stages of maturation, and for the data in Fig. 5A. Pod and seed data from protected or beetle-damaged umbels (Fig. 5B) had unequal variances so were analyzed by the nonparametric Wilcoxon rank sum test. All data analyses were performed using Statistix 10^39^.

## Electronic supplementary material

Supplementary Materials

## Data Availability

All data are presented as means ± SE in the main text. Raw data are available from the corresponding author upon request.
