# Supplementary material for: Japanese beetles’ feeding on milkweed flowers may compromise efforts to restore monarch butterfly habitat
Source: Sci Rep. 2018 Aug 14;8:12139. doi: 10.1038/s41598-018-30731-z (PMC6092377; doi:10.1038/s41598-018-30731-z)
Supplement: Supplementary file 1 — Supplementary Materials [file 41598_2018_30731_MOESM1_ESM.docx]

Supplementary Materials for

Japanese beetles’ feeding on milkweed flowers may compromise efforts to restore monarch butterfly habitat

Adam M. Baker, Daniel A. Potter^.^

Correspondence to: dapotter@uky.edu

**This PDF file includes:**

Supplementary Figs. S1–S3


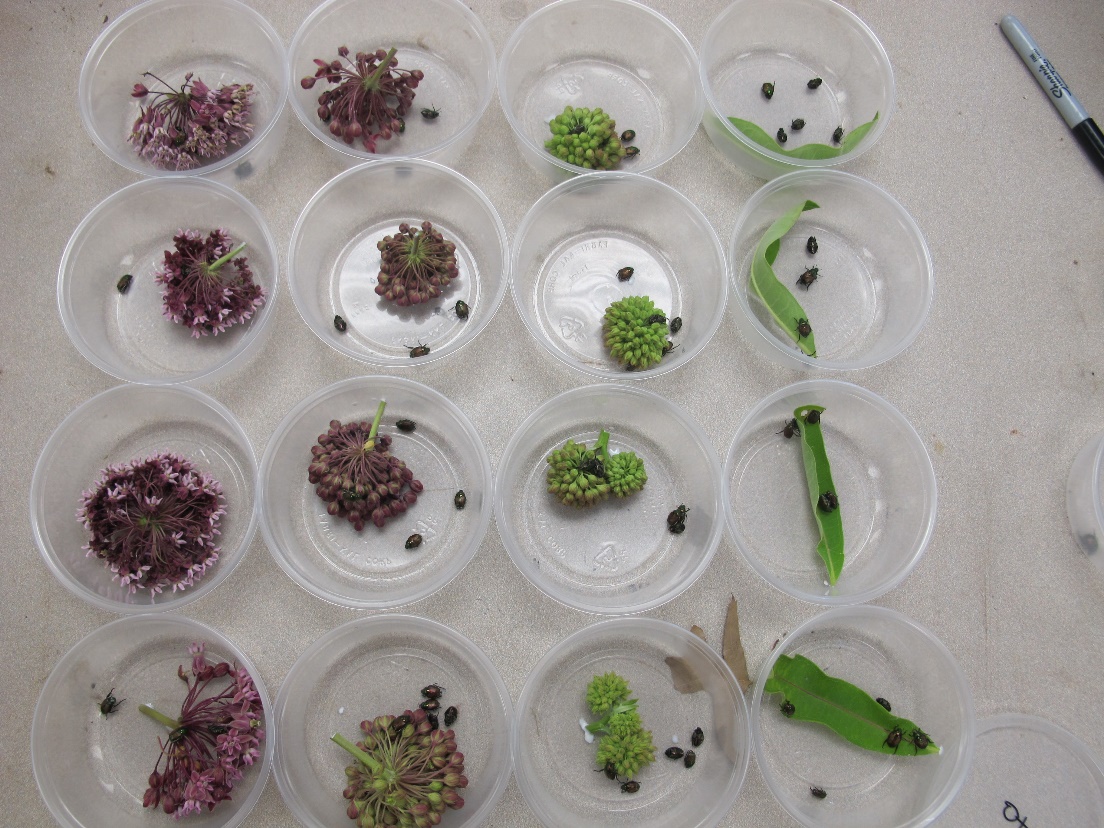


**Supplementary Fig. S1.** To clarify how milkweed flower bud development affects susceptibility to feeding by *P. japonica*, field-collected *Asclepias syriaca* umbels with (left to right) open flowers, pink buds, or closed green buds were confined with five female beetles for 24 h, after which number and percentage of damaged buds or flowers was evaluated.


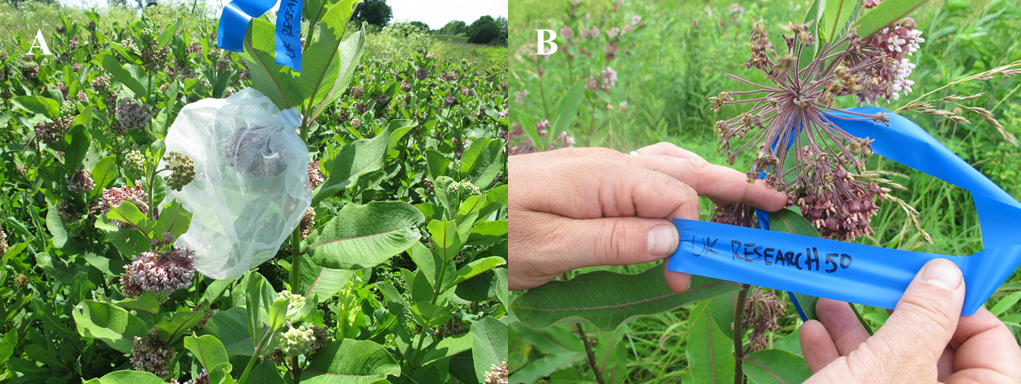


**Supplementary Fig. S2. (A)** Mesh cage enclosing *P. japonica* aggregation on *A. syriaca* umbel. (**B**) Damage to umbel after 24 h feeding by aggregation of 50 beetles.


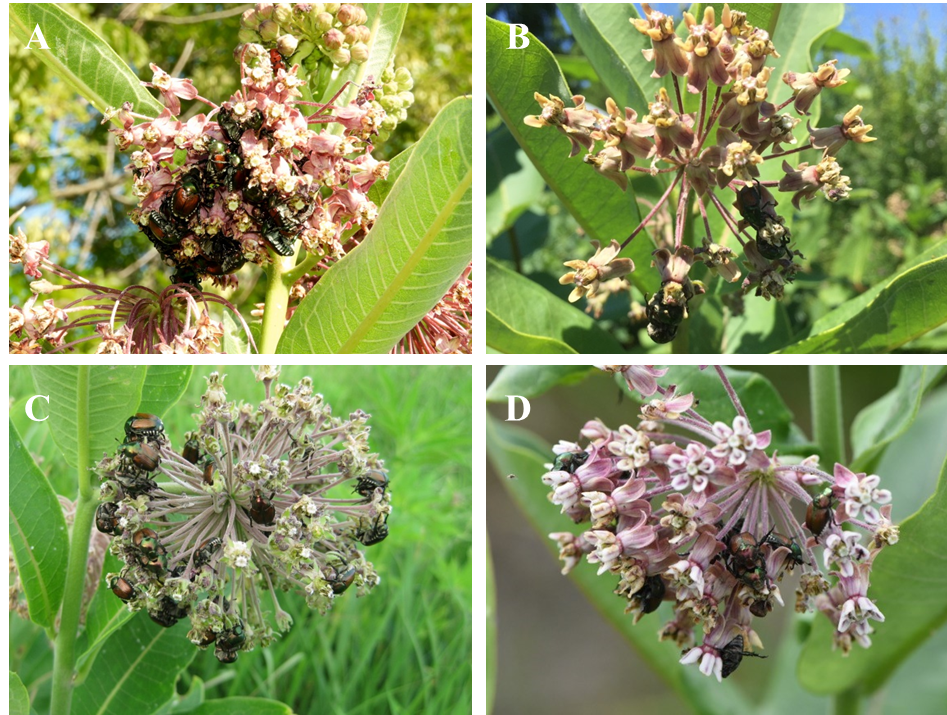


**Supplementary Fig. S3. (A)** *P. japonica* feeding on umbel of *A. syriaca* in Ohio where the beetles have been long established (Photo: C.E. Young). **(B)** Small aggregation of *P. japonica* feeding on milkweed umbel in Minnesota, with damage from nectar-robbing (coronal hoods have been removed to access ovaries and nectaries) (Photo: B. Thilmony). **(C)** and **(D)** Aggregation of *P. japonica* feeding on milkweed umbel in Iowa and Nebraska, respectively, near the invasion front (Photos: L. Iles and T. Weissling).
